# Reflections on the Development and Implementation of a University Student Health and Well-being Online Survey: the BOOST-Well Project

**DOI:** 10.12688/f1000research.168558.4

**Published:** 2026-09-18

**Authors:** Justin Keogh, Evelyne Rathbone, Wendy J Brown, Beth Mozolic-Staunton, Gregory Cox, James Furness, Jaclyn Szkwara

**Affiliations:** 1 Faculty of Health Sciences and Medicine, Bond University, Robina, Queensland, 4227, Australia

**Keywords:** College, health behaviours, questionnaires, recruitment, response rate, Administration, Community Health, Experimental Design, Health Education

## Abstract

**Background:**

First year university students may be at risk of poor health behaviours and outcomes. Unfortunately, online surveys assessing multiple aspects of the health and well-being of university students have poor response rates, meaning the representativeness of such data may be questionable. This study sought to develop and implement an online survey to examine the health and well-being behaviours of medical and allied health students, with higher participation and response rate than have been reported in previous surveys.

**Methods:**

A cross-sectional online survey was developed following recommendations to maximise the participation and response rates. All new students (defined as commencing a degree in May or September 2024) from undergraduate medical and postgraduate allied health programs from one Australian university were requested to participate. The survey included 136 items, most of which were validated questionnaires commonly used in national surveys. Participants were requested to complete the survey on their own device during scheduled class time within the first two weeks of their degree.

**Results:**

Of 273 eligible students, 217 (79.5%) accessed the survey, with 201 (73.6%) completing it at least partially and 63.7% completing it fully. Median completion time was 14.4 (IQR: 12.3–16.8) minutes, and item-level response rates were high across disciplines. Differences in completion rates and survey duration were observed, with occupational therapy students taking the longest time to complete the survey (17.4 min).

**Conclusions:**

The BOOST-Well survey achieved markedly higher response rates than comparable studies. This may reflect the student-informed survey design, concise format, strategic timing, and evidence-based recruitment and implementation strategies.

## Introduction

Lifestyles and health behaviours of students are important determinants of their ongoing health, as well as their academic achievement and future career success. ^1, 2^ However, many students face a variety of physical, emotional, social and academic challenges that may negatively impact their health behaviours and ongoing health and well-being. ^1, 3, 4^ Such challenges may be greater in university than school-age students, as university students may perceive additional study, financial and graduate employability stress, which may be compounded by leaving home, the need for casual/part-time employment and loss of some social connections. ^5– 8^ There is also some evidence demonstrating that these challenges faced by university students may differ based on their country of study ^9^ and their sex. ^10^

Health and well-being information about university students is typically obtained through surveys. However, health survey research traditionally have limitations, including poor reporting of the survey or core questions, questionable validity and reliability of the survey items, poor reporting of the response rate, unclear representativeness of the sample and limited information about how missing data are handled. ^8^ Moreover, response rates to online surveys which seek to assess multiple aspects of the health and well-being of university students are typically low, ranging from 9 to 14% in recent studies. ^3^^,^^8^^,^^11^^,^^12^ A few exceptions report greater but potentially suboptimal response rates, for example 22% for an Australian university-wide survey ^1^ and 31% for a USA-based nursing program. ^13^ Such low response rates are problematic for universities who wish to accurately identify and better manage the health and well-being challenges faced by their students.

By addressing the limitations of survey design, ^14^ university educators, administrators and support service staff may gain a better understanding of the health and well-being challenges of their students. This understanding is an important step in ensuring appropriate resources and services are available and easily accessible by students when required.

The primary aim of this study was therefore to develop and implement an online survey to examine the health and well-being behaviours of medical and allied health students, with higher participation and response rate than have been reported in previous surveys.

## Materials and methods

### Study design and setting

A cross-sectional online survey, **BO**nd **O**nline **S**urvey for **S**tudent Health and **WELL-**being **T**racking (BOOST-Well) survey, was developed and then administered in May or September 2024 at the Faculty of Health Sciences and Medicine (FHSM) at a private Australian university (Bond University). Within this faculty, students from Medicine and several allied health programs (Physiotherapy, Occupational Therapy, and Nutrition and Dietetics) were eligible to participate. These programs were selected because they share the potential for high student stress reflecting students’ contact hours, hospital-based practicums and challenging assessments. Further students in these programs all need to complete research coursework subjects, whereby the inclusion of student survey data could provide an alternative to the systematic reviews or clinical audits typically conducted.

The Consensus-based Checklist for Reporting of Survey Studies checklist (CROSS) was utilised as a framework for conducting and reporting on the survey ^15^ and recruitment methods were informed by the work of Javidan et al. ^16^ Baseline data were collected to inform a planned prospective cohort study of student health and behaviours.

### Survey development

The BOOST-Well survey was developed over 18 months with initial input from 45 students who responded to a single question preliminary survey “What are the five most important issues that affect your health and well-being?” in June 2022. After review of the preliminary survey findings, the authors worked with faculty staff and with student representatives from each program to select and refine the survey questions with formatting to support clear response options.

The BOOST-Well survey was reviewed by three student leaders for the FHSM, the FHSM Community Health and Well-being Officer, one academic from FHSM and all members of the research team, for critical content and layout. Minor changes were made to improve clarity, without altering any wording in previously validated scales. Based on this review, it was estimated it would take the participants 20-25 minutes to complete the survey. The survey is included in Appendix 1.

### Participants and recruitment

During the development of the survey, the authors worked with elected student representatives and staff from each program (Medicine, Physiotherapy, Occupational Therapy and Nutrition and Dietetics). The student representatives were asked to provide some feedback on the topics to include in the survey, potential responses to the questions as well as wording of individual questions and responses to ensure they were appropriate and sensitive to the potential participants. Student representatives also provided advice regarding recruitment strategies, with this advice appearing consistent with many of the recommendations within reviews of previous student surveys ^1^^,^^3^^,^^8^^,^^9^^,^^11^^–^^13^ and the recommendations of Javidan et al. ^16^ on ways to maximise response rates.

All new and enrolled students (defined as commencing a degree that semester) from the undergraduate medical and postgraduate allied health programs were invited to participate. Students completed the online survey through Qualtrics ( https://www.qualtrics.com/) on their own device (laptop or phone) during program specific class time in Orientation Week (O-Week), the week prior to formal classes commencing, or within the first two weeks of commencing their degree. Scheduling for each group was based on maximising the expected number of students who would attend the selected class. Physiotherapy and Nutrition and Dietetics students completed the survey in May 2024 and Occupational Therapy and Medical students in either May or September 2024. To minimise coercion, lead research academics, who were not directly involved in the specific program in which each group of students was enrolled, presented ‘in person’ PowerPoint slides alongside a video to explain the aims and rationale of the study. The PowerPoint presentation typically lasted 5-10 minutes and focused on issues related to informed consent as well as how issues such as data privacy and confidentiality, minimising harm and maximising benefits of participation to themselves and future students would reflect their involvement in the study. It was also made explicitly clear to the students that their participation was voluntary and that there was no requirement for them to complete the survey, or that their decision not to participate would negatively influence their grades in any way. A QR code was provided for participants to access the survey and to enter a draw for an incentive on completion, with randomly selected students winning one of twenty $100 AUD gift cards. Only students who were present in class at the time the PowerPoint presentation describing the survey was given were eligible to participate in this study i.e. students who did not attend class on their respective days could not participate in the study.

### Statistical analysis

Descriptive statistics are presented as counts and percentages for categorical variables. For continuous variables, normality was assessed using histograms, normal Q-Q plots and the Shapiro-Wilk test. Skewed variables are reported as (medians with IQR). Differences in categorical variables between study programs were compared using the chi-square test, provided the assumption for expected counts was met. The non-parametric Kruskal-Wallis test was used to assess program differences in skewed continuous variables. Statistical significance was set at the 0.05 level. All analyses were conducted using Jamovi software version 2.3.28. ^17^

## Results

The five most important issues identified by students in the preliminary survey in 2022 were stress (study and financial), time pressure, social support, general health (physical and mental health), and healthy lifestyle (nutrition, sleep, smoking and alcohol consumption). These issues were incorporated into the BOOST-Well survey, which included questions in six groups, with a total of 136 items. These included demographic characteristics (as used by the Australian Bureau of Statistics ^18^) and wherever possible, validated questions or scales which have been used in national surveys in Australia, or in prior surveys of university students. When no suitable measures were found, the authors developed or modified questions, for example in relation to the students’ top three health concerns, swimming ability, training in life saving, first aid and resuscitation. Items included in each section of the survey, with sources and response rates, are shown in Table 1.

**Table 1.** Summary of survey content and item response rates for the 136 survey variables (based on valid cases, *N *= 201).

Survey section	Variable/measure or scale	Reference	Items (#)	Response rate (%)
Demographic characteristics	Age	Adapted from ABS census ^18^	1	**88.6**
	Gender	Adapted from ABS census ^18^	1	**100.0**
	Country of birth	Adapted from ABS census ^18^	1	**100.0**
	Language usually spoken at home	Adapted from ABS census ^18^	1	**99.5**
	Student status	Internal university item	1	**100.0**
	Indigenous origin	ABS census ^18^	1	**99.5**
	Highest qualification	Adapted from ABS census ^18^	1	**100.0**
	Program of study	Internal university item	1	**100.0**
	Living arrangements	ABS census ^18^	1	**99.5**
	Employment status	Adapted from ABS census ^18^	1	**99.5**
	Income management	ALSWH ^19^	1	**99.5**
	Postcode	Adapted from ABS census ^18^	1	**99.5**
Quality of Life (physical and mental health)	SF-12 version 1 (standard) for physical and mental health	Ware et al. ^20^	12	**94.0**
	Top 3 health concerns	Self-developed	1	56.2 ^a^
	Kessler K10 mental health scale	Kessler et al. ^21^	10	93.5 – 94.0
Time use, stress and social support	Time management, use of time, and amount of time that work/study affected physical and emotional well-being	ALSWH ^19^	5	75.1 – 92.0
	Stress	Bell and Lee ^22^	11	92.0
	MOS social support	Sherbourne and Stewart ^23^	19	91.0 – 92.0
Health behaviours	Smoking, vaping and alcohol consumption	Based on or adapted from ALSWH ^19^	13	53.3 ^b^ – 100
	Physical activity	Modified Active Australia survey ^24^	8	**85.6**
	Muscle strengthening	Adapted from NHS ^25^	2	>86.0
	Transport	Modified from HABITAT ^26^	2	~86.0
	Swimming ability	Self-developed	3	~86.0
	Training in life saving, first aid and/or resuscitation	Self-developed	6	67.0 – 78.6
	Sedentary behaviour	Chau et al. ^27^ & Clark et al. ^28^	2	77.6
	Fruit and vegetable consumption	NHS ^25^	2	~86.0
	Diet and meals bought	Adapted from NHANES ^29^	4	~86.0
	Height and weight	Adapted from NHS ^25^	2	81.6 – 84.6
	Sun protection	ALSWH ^19^	6	~86.0
Health services and medications	Visits to health professionals	ALSWH ^19^	13	~86.0 ^c^
	Medications and supplements	ALSWH ^19^	2	22.9 – 32.3 ^a^

**Note:** Values in bold font are confirmed; values in regular font are estimated.

**Abbreviations:** ABS Australian Bureau of Statistics; ALSWH Australian Longitudinal Study on Women’s Health; HABITAT How Areas in Brisbane Influence healTh And acTivity; MOS Medical Outcomes Study; NHANES National Health and Nutrition Examination Survey; NHS National Health Survey; SF Short Form.

^a^ Qualitative data unlikely to apply to all students.

^b^ Percentage based on applicable cases (only 8 of 15 smokers or ex-smokers gave the age when they started smoking).

^c^ Rate based on items excluding question about any other health professional seen.

In consultation with our student representatives, and the recommendations of Javidan et al., ^16^ recruitment strategies adopted for the BOOST-Well survey included: Faculty and ‘in-kind’ support in terms of staff time, space, and IT resources, (Recommendation #1); student input throughout the survey development process (Recommendation #3); Faculty budget support for incentives (20 x $100 AUD gift cards for the student group) (Recommendation #5); and generation of student awareness in the form of a promotional video which was developed to ensure that consistent information was provided to each group of students before they completed the survey (Recommendation #6). These strategies are compared with those used in seven earlier student surveys ^1, 3, 8, 9 11– 13^ in Table 2. These seven studies are a non-exhaustive list of studies published since 2015 that report survey-based health and well-being data from university students.

**Table 2.** Comparisons of recruitment strategies and response rates in previous student surveys based on the six recommendations of Javidan et al. ^16^

Author date and place	Participants	Response numbers	Response rate (%)	Time to complete	Javidan et al. ^16^ six recommendations					
					Faculty support	Assigned student reps	Incorporated participant input into survey design	Protected time in class to complete survey	Incentives offered	Generated student awareness
Fruh et al., ^13^ 2021 USA	Undergraduate Nursing, 570 students	176 completed	31%	NR	Faculty supported recruitment processes; provided access to Qualtrics survey and statistical software; provided financial incentives for survey completion	NR	NR	NR	$15 eligible for electronic gift card	Initial email for distribution; 7 automated email reminders
Holt and Powell, ^12^ 2017 UK	University wide, available online to 32,000 students	3683 commenced (3428 completed)	11%	NR	Faculty supported recruitment processes; provided access to Qualtrics survey and statistical software	NR	Engaged student services to inform included questions	NR	NR	Initial email for distribution
Reichel et al., ^11^ 2021 Germany	University wide, available online to 31,213 students	4714 commenced (4351 completed)	14%	Estimated 35–45 minutes	Faculty supported recruitment emails; provided access to physical spaces for participant recruitment and survey completion, Unipark survey and statistical software; provided financial incentives for survey completion	NR	12 students completed a pre-test, minor adjustments made thereafter	NR	Incentives provided, fresh fruit @ physical space; charitable donation if > 5000 students completed survey (1000€), individual gift cards (13 x 24-40€) for local restaurants and for online store (15 x 20-100€)	Initial email for distribution; 4 reminder emails; research team members attended lectures; lecturers included slides; promotional material – posters, leaflets, newspaper press release, social media
Sanci et al., ^1^ 2022 Australia	University wide, available online to 56,375 students	14,880 commenced (12,347 completed)	22%	Estimated 20 minutes	Faculty supported recruitment processes; provided access to Qualtrics survey and statistical software; provided financial incentives for survey completion	NR	The project team was Advised by a stakeholder advisory group including student association; pilot tested in a 4h workshop with 15 students. Students provided feedback on framing and comprehension of questions, survey length and item order	NR	Random draw >50 prizes (ipads, cycle vouchers, gift cards)	Initial email distribution; 2-weeks prior posters, flyers, digital slides for lecturers, online student social media channels, promotional video; reminder emails weekly – 8 weeks
Skromanis et al., ^3^ 2018 Australia	University wide, available online to 15,259 students	1,013 AUS 382 INT	9% 9%	Estimated 20 minutes	Faculty supported recruitment processes; provided access to survey and statistical software; provided financial incentives for survey completion	NR	Pilot study to elicit feedback	NR	Gift vouchers, value not reported	Initial email distribution; single reminder email & SMS; social media, flyers and postcards
Whatnall et al., ^8^ 2019 Australia	University wide, available online to 33,783 students	3,529 commenced (3077 completed); Optional questions: 3025 drug use; 1786 sexual health; 2962 mental health	9%	Estimated 15 minutes plus optional sensitive questions on drug use, sexual health and mental health	Faculty supported recruitment processes; provided access to Survey Monkey survey and statistical software; provided financial incentives for survey completion	NR	NR	NR	Gift vouchers (5 x $100 AUD)	Bulk email distribution; 2 reminder emails; university staff prompted to promote the survey; social media; digital signage; posters
Yeh et al., ^9^ 2023 Australia and Taiwan	Nursing, available via pen and paper to an unknown number of eligible students	381 completed survey (201 Australian, 180 Taiwanese)	NR	Estimated 30 minutes	Faculty supported recruitment processes; provided students access to hardcopy questionnaires and pencils survey; financial incentives for survey completion	NR	NR	NR	$2 chocolate	Verbal explanation by researchers during class; written material provided to students in class
Number of studies following Javidan’s Recom					7 of 7	0 of 7	4 of 7	0 of 7	6 of 7	7 of 7

NR = not reported, Recom = recommendations, Reps = representatives.

Data on survey completion rates and time taken to complete the survey are shown in Table 3.

**Table 3.** Summary of survey completion rates and times taken to complete the survey for the overall cohort and each discipline group.

	Total( *N *= 201)	Medicine ( *n* = 114)	Physiotherapy ( *n* = 48)	Occupational therapy ( *n* = 27)	Nutrition & dietetics ( *n* = 12)	Group differences (p-value)
** Survey completion**	N %	n %	n %	n %	n %	NR
Partial	27	22	1	3	1	
	13.4	19.3	2.1	11.1	8.3	
Complete	174	92	47	24	11	
	86.6	80.7	97.9	88.9	91.7	
**Duration (mins) of fully completed survey**	Median	Median	Median	Median	Median	
	IQR	IQR	IQR	IQR	IQR	
	14.4	14.4	14.2	17.4	16.8	.006 *
	12.3–16.8	12.1–16.2	12.3–16.3	13.4–22.2 ^a^	14.2–19.7	

Percentages based on valid cases. NR = Not Reported (due to low counts in some cells).

^*^ Statistically significant <.05.

^a^ Significantly shorter in Medicine ( *p *= .016) and Physiotherapy ( *p *= .042) groups.

Of 273 registered students, 217 (79.5%) viewed the initial section of the online questionnaire, which preceded the actual survey questions. Of these, 201 proceeded to either fill out the survey, (either partially ( *n* = 27; or completely ( *n* = 174)), for an overall response rate of 73.6% (201/273) at least partial completions and 63.7% full completions. Full completion was defined at the participant level as completing all questions within the survey; with partial completion being defined as completing at least all the demographic information questions. Completion rates ranged from 80.7% to 97.9% for individual programs, with medical students having the lowest completion rate. The median (IQR) time taken for completion was 14.4 (12.3-16.8) minutes.

The Occupational Therapy students took significantly longer to complete the survey than the medicine or physiotherapy students (see Table 3).

Response rates to each section of the survey were high, but missing data were common in questions which did not apply to some individuals or required students to recall events that may have occurred more than two months before the survey (e.g., age when started smoking, year of completing resuscitation training). Response rates to open-ended questions, such as ‘top 3 health concerns’ and ‘medications and supplements’ were also low (see Table 1).

A summary of participants’ demographic characteristics is provided in Table 4 for the total sample and for students in each of the four program groups. There were significant group differences in age and language spoken at home. Medical students were younger than students in the three allied health programs and the Occupational Therapy and Nutrition and Dietetics students were less likely to speak English at home than the other students.

**Table 4.** Demographic and study enrolment characteristics of participants.

Characteristics	Programs				
	All ( *N* = 201)	Medicine ( *n* = 114)	Physiotherapy ( *n* = 48)	Occupational Therapy ( *n* = 27)	Nutrition and Dietetics ( *n* = 12)
**Age (years)**, median (IQR)	20.5 (18–25)	18 (18–19) ^a^	25 (24–27)	24 (23–30)	25 (21.8–26)
Range	18–48	18–31	21–48	19–39	20–48
Missing, *n* (%)	23 (11.4)	14 (12.3)	8 (16.7)	1 (3.7)	0 (0.0)
**Gender**, *n* (%)					
A woman	130 (64.7)	70 (61.4)	27 (56.2)	22 (81.5)	11 (91.7)
A man	69 (34.3)	43 (37.7)	21 (43.8)	4 (14.8)	1 (8.3)
Prefer not to say/Other	<5%	<5%	<5%	<5%	<5%
**Indigenous origin**, *n* (%)					
No	200 (100)	113 (100)	48 (100)	27.0 (100)	12 (100)
Missing	1 (0.5)	1 (0.9)	0 (0.0)	0 (0.0)	0 (0.0)
**Country of birth**, *n* (%)					
Australia	83 (41.3)	65 (57.0)	10 (20.8)	6 (22.2)	2 (16.7)
Other English-speaking country	49 (24.4)	18 (15.8)	25 (52.1)	4 (14.8)	2 (16.7)
Non-English-speaking country in Asia	58 (28.9)	26 (22.8)	11 (22.9)	17 (63.0)	4 (33.3)
Other	11 (5.5)	5 (4.4)	2 (4.2)	0 (0.0)	4 (33.3)
**Language usually spoken at home**, *n* (%)					
English	129 (64.5)	78 (69.0)	38 (79.2)	9 (33.3)	4 (33.3)
Other	71 (35.5)	35 (31.0)	10 (20.8)	18 (66.7)	8 (66.7)
Missing	1 (0.5)	1 (0.9)	0 (0.0)	0 (0.0)	0 (0.0)
**Living arrangements**, *n* (%)					
Live alone	39 (19.5)	22 (19.3)	7 (14.9)	7 (25.9)	3 (25.0)
On campus – shared	68 (34.0)	60 (52.6)	5 (10.6)	3 (11.1)	0 (0.0)
Off campus – shared	60 (30.0)	19 (16.7)	28 (59.6)	9 (33.3)	4 (33.3)
Other	33 (16.5)	13 (11.4)	7 (14.6)	8 (29.6)	5 (41.7)
Missing	1 (0.5)	0 (0.0)	1 (2.1)	0 (0.0)	0 (0.0)
**Income source**, *n* (%)					
No paid work	106 (53.0)	54 (47.4)	32 (68.1)	15 (55.6)	5 (41.7)
Regular paid work	55 (27.5)	30 (26.3)	8 (17.0)	10 (37.0)	7 (58.3)
Irregular paid work	39 (19.5)	30 (26.3)	7 (14.9)	2 (7.4)	0 (0.0)
Missing	1 (0.5)	0 (0.0)	1 (2.1)	0 (0.0)	0 (0.0)
**Highest qualification**, *n* (%)					
School only	96 (47.8)	96 (84.2)	0 (0.0)	0 (0.0)	0 (0.0)
Bachelor’s degree	86 (42.8)	13 (11.4)	44 (91.7)	19 (70.4)	10 (83.3)
Other	19 (9.5)	5 (4.4)	4 (8.3)	8 (29.6)	2 (16.7)
**Level of study**, *n* (%)					
Undergraduate	114 (56.7)	114 (100.0)			
Postgraduate	87 (43.3)		48 (100.0)	27 (100.0)	12 (100.0)

Percentages were calculated from valid cases. Percentages for missing values were based on total within group.

^a^ Significantly different from all other groups (p < .001 for all post-hoc pairwise comparisons).

A summary of the data is available on the project’s Open Science Framework page. ^36^

## Discussion

The primary aim of this study was to develop and implement an online survey to examine the health and well-being behaviours of medical and allied health students, with higher participation and response rates than have been reported in previous surveys. As the survey data will be used to inform the development of evidence-based and targeted health promotion strategies, and as a resource for students to develop research skills in data management and analysis within their research subjects, it was important to achieve a high response rate across all the health professional programs. The completion rates of 73.6% (at least partial completion) and 63.7% (full completion) were markedly higher than those reported in previous studies in Australia, ^3, 8^ Germany ^11^ and the UK ^12^ that typically had response rates of 9-14%. There are however a small number of recent studies with higher response rates, including one Australian university-wide study that had a response rate of 22% ^1^ and a USA based study that recruited only nursing students with a response rate of 31%. ^13^

The higher response rate for the BOOST-Well survey may be explained by several factors; many of which overlap with the six strategies recommended by Javidan et al. ^16^ as being critical for maximising response rates to student surveys.

We involved students throughout the survey development process to inform survey content, recruitment strategies and incentives. Reichel et al. ^11^ and Sanci et al. ^1^ (who obtained response rates of 14% and 22% respectively to university-wide surveys), also incorporated some student input into their survey development. Incentives have been widely used in previous studies, usually in the form of gift vouchers with value between $15 and $100. ^3^^,^^8^^,^^11^^,^^13^ Two recent meta-analyses have concluded that appropriate incentives for maximising response rates to online surveys are unclear, ^30, 31^ so we cannot say whether our incentive approach affected our response rate. Our use of a video to explain the survey on the day of completion precluded the need for initial or follow-up reminder emails, or promotional materials such as posters, slides, or social media, as was the case in previous studies. ^1^^,^^3^^,^^8^^,^^11^^,^^13^

A major difference between our approach and that used in previous studies is that we provided protected time in class for students to complete the survey. The only similar approach was by Yeh et al., ^9^ who provided hard copy surveys to their students during class-time but required them to complete the survey in their own time. Our students completed the survey in class time during Orientation week or within the first two weeks of commencing their degree. At this time, they were new to the university and were not overly encumbered with classes and assessments, nor other requests to complete formal feedback surveys for the university (i.e. teaching evaluations). The survey was also kept as short as possible, so that completion time would be minimised. The median completion time (14.4 minutes) was a little shorter than anticipated and substantially shorter than most previous surveys, which often took 20-45 minutes to complete. ^1, 3, 8, 11^ A review by Sammut et al. ^32^ indicates that short surveys of ~10 minutes have substantially better response rates than longer surveys; the brevity of the survey may therefore have positively influenced our response rate. Others have shown that survey length and the complexity of individual questions, as well as the percentage of open-ended questions, may be related to reduced response rates and greater amounts of missing data. ^33^

Overall, the combination of codesigned survey development and recruitment strategies, which align well with those proposed by Javidan et al. ^16^ probably underpin the high response rates to the BOOST-Well survey. However, it is acknowledged that Bond University is a small institution with a strong culture of student engagement, small class sizes and personalised teaching. ^34^ This, together with the focus on health professional students, may help to explain the strong response rate.

### Strengths and limitations

A major strength of this study was the student-informed and co-designed development of the survey methodology. This process incorporated input from both students and staff across multiple programs within a single faculty and aligned closely with the recommendations of Javidan et al. ^16^ As survey development requires a balance between comprehensiveness and conciseness, and the need to minimise completion time, our survey focused on health and well-being topics that an earlier pilot test of 45 of our students felt were most important. This meant that some health issues were not included. While this is a limitation, new issues, such as alternative dietary patterns, sleep, social media and/or screen use and reproductive health may be included in follow-up surveys. As the entire population of newly registered students in these programs was only 273, we also need to acknowledge the small sample size and lack of power analysis. Partially due to the small sample size, another limitation of the BOOST-Well surveys is that the external validity of this study in that the health priorities identified by Bond students may not be applicable to students from larger public institutions.

A further limitation is that completing the survey during class time may have created perceived participation or social desirability bias pressure, and some students may have felt less privacy when seated near classmates. We sought to minimise these risks by emphasising that participation was voluntary, that their answers should reflect their own opinions, that non-participation would have no academic consequences, and that they should not view others’ responses. Most students completed the survey on personal smartphones, which likely helped protect privacy by further reducing the potential that classmates could see each other’s responses.

### Next steps

The BOOST-Well data will be used to develop targeted strategies for improving student health and well-being. The data will also be used as a resource for students to learn about data cleaning, coding and statistical analysis. This is important because it is challenging to access ‘real world’ data for development of research skills, because of time restrictions associated with obtaining ethical clearance for collection of data in a single trimester. Educators will now encourage students to work in interprofessional groups, to provide meaningful insights for discussion and reflection of student health issues from an interdisciplinary perspective. ^35^ In future, it is planned to evaluate student perspectives on their involvement in this project and assess whether the project is useful for development of research interests, literacy and skills and interprofessional practice skills.

## Conclusion

By following a series of recommendations from the literature, we developed an online health and well-being survey that had a completion rate that is substantially higher than that typically reported for other studies involving university students. The data will inform the future development of evidence-based and targeted strategies for improvement of students’ health and well-being, and as a resource for students to develop research skills.

## Ethical considerations

A data custodian team was created to establish secure data storage and processing protocols, with the faculty statistician as the lead data custodian. To ensure that data from individual respondents could not be identified in subsequent analyses, each participant created a self-generated identification code (SGIC) before completing the survey. SGICs were based on elements of personal information known only to the student, in order to enable effective longitudinal tracking, should the survey be completed again in the future by the same students. ^36^ Data linked to the SGICs were initially extracted from Qualtrics and saved in a separate data store, accessible only to the lead data custodian, who then created a new identifier code to replace the SGIC created by individual students. The SGIC code elements were re-ordered and recoded according to a mapping system created by the lead data custodian, with details in a password protected file that is only accessible by the lead data custodian. Once all potentially identifying variables were removed, data were transferred to a separate data store for use by members of the research team. Participants provided informed consent electronically prior to completing the online survey through Qualtrics. The study adhered to the Declaration of Helsinki and was approved by the Bond University Human Research Ethics Committee (JK02927).

## Data Availability

Open Science Framework: BOOST-Well: **BO**nd **O**nline **S**urvey for Student Health and **Well**-being **T**racking, https://doi.org/10.17605/OSF.IO/DHQBY ^37^ This project contains the following underlying data: 2024_May_BOOST_data_201.xlsx Data are available under the terms of the Creative Commons Attribution 4.0 International license (CC-BY 4.0). Open Science Framework: BOOST-Well: **BO**nd **O**nline **S**urvey for Student Health and **Well**-being **T**racking, https://doi.org/10.17605/OSF.IO/DHQBY ^37^ This project (2024_May_BOOST_data) contains the following underlying data: 2024_May_BOOST_data_201.xlsx **Multimedia Appendix 1.pdf** Data are available under the terms of the Creative Commons Attribution 4.0 International license (CC-BY 4.0).
