# Supplementary material for: Reflections on the Development and Implementation of a University Student Health and Well-being Online Survey: the BOOST-Well Project
Source: F1000Res. 2026 Sep 18;14:808. Originally published 2025 Aug 20. [Version 4] doi: 10.12688/f1000research.168558.4 (PMC13616052; doi:10.12688/f1000research.168558.4)
Supplement: Peer review history

# Reviewer report: Reviewer response for version 4

**DOI:** 10.5256/f1000research.210716.r522394

**Published:** 2026-10-10

**Authors:** Ali Azeez Al-Jumaili

**Affiliations:** 1 Department of Clinical Pharmacy, University of Baghdad, Baghdad, Baghdad Governorate, Iraq

I would like to thank the authors for addressing all of my previous comments. I have no further comments.

Is the work clearly and accurately presented and does it cite the current literature?

No

If applicable, is the statistical analysis and its interpretation appropriate?

Partly

Are all the source data underlying the results available to ensure full reproducibility?

Partly

Is the study design appropriate and is the work technically sound?

Partly

Are the conclusions drawn adequately supported by the results?

No

Are sufficient details of methods and analysis provided to allow replication by others?

No

Reviewer Expertise:

Pharmaceutical Socioeconomics, Pharmaceutical/Medical Education, Clinical and Social Pharmacy, Pharmacy Practice, Pharmacy Policy, and Pharmacoeconomics.

I confirm that I have read this submission and believe that I have an appropriate level of expertise to confirm that it is of an acceptable scientific standard.

# Reviewer report: Reviewer response for version 4

**DOI:** 10.5256/f1000research.210716.r522392

**Published:** 2026-09-26

**Authors:** Hengki Yandri

**Affiliations:** 1 Institut Agama Islam Negeri Kerinci, Jambi, Indonesia

I have assessed the revisions made by the authors based on the previous review report, I am pleased to inform that the revisions are appropriate and fully address my previous comments. Therefore, I would like to change the status of this article to Approved.

Is the work clearly and accurately presented and does it cite the current literature?

Partly

If applicable, is the statistical analysis and its interpretation appropriate?

Yes

Are all the source data underlying the results available to ensure full reproducibility?

Yes

Is the study design appropriate and is the work technically sound?

Yes

Are the conclusions drawn adequately supported by the results?

Partly

Are sufficient details of methods and analysis provided to allow replication by others?

Partly

Reviewer Expertise:

Spiritual Counseling, Counseling, Educational Psychology, and Mental Health

I confirm that I have read this submission and believe that I have an appropriate level of expertise to confirm that it is of an acceptable scientific standard.

# Reviewer report: Reviewer response for version 3

**DOI:** 10.5256/f1000research.199248.r491959

**Published:** 2026-09-11

**Authors:** Clodagh Flinn

**Affiliations:** 1 University College Dublin, Dublin, Ireland; 2 Psychology, Carleton University (Ringgold ID: 6339), Ottawa, Ontario, Canada

**General Comment**

 Thank you for inviting me to review this article again. I believe that the authors have greatly improved this manuscript. I believe that the integrity of this manuscript is sound, and it can provide useful information for researchers on how to increase participation in research surveys. There remain, however, several points which should be addressed before it can go to indexation. Some of these were raised in previous versions of the manuscript, but I do not feel they have been comprehensively addressed yet. However, I believe that the integration of the points below will improve clarify and aid interpretation of this interesting and beneficial study. A point-by-point response to the review comments would be useful.

**Specific Comments**

**Introduction:**

 - The justification for including students in the study is presented well. However, further justification for the inclusion of medical and allied health programmes specifically is needed and will provide important context for the study. This was raised in my previous review of this manuscript. Please add this at the end of the first paragraph.

**Method:**

 - Appendix 2 is still not available to view.

 - Can you provide some more information about how the student representatives involved in developing recruitment strategies selected? (Perhaps this information is in Appendix 2?).

 - Thank you for adding more information about how/when the participants completed the survey. In line with reviewer 5, please add a few sentences in the limitations section about how completing the survey during class time may have created participation pressure for.

 Further, is it possible that some participants may have felt a reduced sense of privacy to complete the survey if they were sitting in a lecture hall among their peers? This may have resulted in a social desirability bias. Was this considered? If so, how? 

 - My previous point about how absent students were dealt with (e.g., whether they were provided another opportunity to participate, etc.) has not been addressed. This has also been raised by reviewer 4.

**Results:**

 - How were the seven earlier student surveys presented in Table 2 selected?

 - Small frequencies remain in Table 4 which may put the anonymity of participants at risk, particularly given that the name of the institution and specific course names are provided (e.g., one "man" in the Nutrition and Dietetics course).

 This point has been raised in response to previous versions of the manuscript and by other reviewers. Please ensure that the appropriate measures are taken to protect participants' identities, and are applied consistently across the ***entire *** table.

**Discussion:**

 - Add explicit acknowledgment of the participation pressure and social desirability bias due to completing the survey during class time into the limitations section.

Is the work clearly and accurately presented and does it cite the current literature?

Partly

If applicable, is the statistical analysis and its interpretation appropriate?

Yes

Are all the source data underlying the results available to ensure full reproducibility?

Yes

Is the study design appropriate and is the work technically sound?

Yes

Are the conclusions drawn adequately supported by the results?

Yes

Are sufficient details of methods and analysis provided to allow replication by others?

No

Reviewer Expertise:

Psychological wellbeing and resilience. Chronic health conditions. Developmental transitions.

I confirm that I have read this submission and believe that I have an appropriate level of expertise to confirm that it is of an acceptable scientific standard, however I have significant reservations, as outlined above.

# Response

**Published:** 2026-09-15

**Authors:** JUSTIN Keogh

**Affiliations:** Bond University Faculty of Health Sciences and Medicine, Gold Coast, Queensland, Australia

**General Comment**

 Thank you for inviting me to review this article again. I believe that the authors have greatly improved this manuscript. I believe that the integrity of this manuscript is sound, and it can provide useful information for researchers on how to increase participation in research surveys. There remain, however, several points which should be addressed before it can go to indexation. Some of these were raised in previous versions of the manuscript, but I do not feel they have been comprehensively addressed yet. However, I believe that the integration of the points below will improve clarify and aid interpretation of this interesting and beneficial study. A point-by-point response to the review comments would be useful.

**Response:** **we thank the reviewer for the positive comments regarding some aspects of the manuscript as well is identifying other areas for further improvement.**

**Specific Comments**

**Introduction:**

 - The justification for including students in the study is presented well. However, further justification for the inclusion of medical and allied health programmes specifically is needed and will provide important context for the study. This was raised in my previous review of this manuscript. Please add this at the end of the first paragraph.

**Response: some additional information has been provided as requested. However, it was felt best not to include this at the end of the first paragraph in the introduction but in the first paragraph of the section entitled Materials and Methods / Study Design and Setting, as that is where we first mentioned the faculty from which these students were recruited.**

**In this section, we have now provided some additional detail regarding how the inclusion of these programs reflected a combination of commonalities between these programs in terms of: 1) the students’ high contact hours, hospital-based practicums and challenging assessments that could adversely affect their physical and mental health; and 2) coursework research subjects that are contained in these degrees in which student-focused survey data might provide interesting alternatives to the systematic reviews and clinical audits commonly performed by the students in these programs.**

**Method:**

 - Appendix 2 is still not available to view.

**Response: we apologise for this mistake, with Appendix 2 now being included in the main manuscript as requested by a previous reviewer and labelled Table 2.**

 Can you provide some more information about how the student representatives involved in developing recruitment strategies selected? (Perhaps this information is in Appendix 2?).

**Response: some additional information is now provided regarding the student’s involvement in the recruitment strategies in the Participants and recruitment section within Materials and methods.**

 - Thank you for adding more information about how/when the participants completed the survey. In line with reviewer 5, please add a few sentences in the limitations section about how completing the survey during class time may have created participation pressure for.

**Response: some additional detail has been provided in the Limitations section regarding the limitations of our in-class recruitment process.**

 Further, is it possible that some participants may have felt a reduced sense of privacy to complete the survey if they were sitting in a lecture hall among their peers? This may have resulted in a social desirability bias. Was this considered? If so, how? 

**Response:  while it is possible that some participants may have some reduced sense of privacy, the students routinely participate in a range of online surveys in class assessing their understanding of the subject content. In these typical classroom situations as well is this online survey, the students complete the surveys on personal smartphones and not on classroom computer screens, further reducing any uneasiness about privacy would be minimal, if any. It was also made clear to the participants that they should not look at their classmates responses.**

 - My previous point about how absent students were dealt with (e.g., whether they were provided another opportunity to participate, etc.) has not been addressed. This has also been raised by reviewer 4.

**Response: any students who were absent from class on the day the survey was to be completed were not eligible to be included in the survey. This information is provided at the end of the Participants and recruitment section within Materials and methods.**

**Results:**

 - How were the seven earlier student surveys presented in Table 2 selected?

**Response: the studies were identified from our reading of the literature and database searches using keywords that included “university or college”, “student” and “health survey”, with a date range between 2015 and present, so to ensure that such studies’ results are likely still to be relevant to university students studying today. It has now been made more explicit that these seven studies is not an exhaustive list, but merely an example of the research conducted in this area since 2015.**

 - Small frequencies remain in Table 4 which may put the anonymity of participants at risk, particularly given that the name of the institution and specific course names are provided (e.g., one "man" in the Nutrition and Dietetics course).

 This point has been raised in response to previous versions of the manuscript and by other reviewers. Please ensure that the appropriate measures are taken to protect participants' identities, and are applied consistently across the ***entire *** table.

**Response: we agree that protecting participant confidentiality is important and feel that we have addressed this comment already. Whilst the results table might still contain some lower frequencies, we have taken all necessary steps to protect any potential identification. Other than a single person (the data custodian not linked to the medical or allied health programs) who produced the results for this table, no one else had access to any information related to the small group of individuals since all the IDs collected anonymously were recoded and participants from programs (physiotherapy, occupational therapy, and nutrition and dietetics) were merged into one subgroup called ‘Allied Health’ prior to the data being made available to anyone else, so that no particular individuals from the smaller allied health programs can be identified. This de-identified data set in which each individual is categorised as “Medicine” or “Allied Health” can be found in the appendices (2024_May_BOOST_data_201.xlsx)**

**Discussion:**

 - Add explicit acknowledgment of the participation pressure and social desirability bias due to completing the survey during class time into the limitations section.

**Response: this has been done as requested.**

# Reviewer report: Reviewer response for version 3

**DOI:** 10.5256/f1000research.199248.r507586

**Published:** 2026-08-25

**Authors:** Shaimaa Ezzat Basha

**Affiliations:** 1 Psychology, Fujairah University, Fujairah, Fujairah, United Arab Emirates

I have reviewed Version 3 of the article "Reflections on the development and implementation of a university student health and well-being online survey: the BOOST-Well project."

 The manuscript is scientifically sound overall and addresses an important methodological issue: how to improve participation and response rates in online health and well-being surveys among university students. The study is clearly framed as a cross-sectional online survey conducted among new medical and allied health students at one Australian university. The authors provide useful details on survey development, student-informed design, recruitment during scheduled class time, use of validated items where possible, and strategies intended to maximize response and completion rates.

 The article has several strengths. First, the survey development process was informed by student input and relevant survey-design recommendations. Second, the recruitment strategy was practical and well described, including use of scheduled class time, clear consent procedures, and an incentive draw. Third, the reported participation and completion rates are meaningfully higher than those reported in many comparable online student health surveys. Fourth, the authors provide data availability information and ethical approval details, which strengthen transparency and reproducibility.

 However, I still have some reservations.

 1. The manuscript should more clearly distinguish between the aim of the survey itself and the aim of the current article. The article states that the survey was designed to examine student health and well-being behaviors, but the manuscript mainly reports survey development, implementation, response rates, and completion time. If the health and well-being outcomes will be reported separately, this should be stated more explicitly in the Introduction, Results, and Conclusion.

 2. The possibility of implicit participation pressure should be acknowledged more directly as a limitation. Although the authors state that participation was voluntary and would not affect grades, completing the survey during scheduled class time may still have increased perceived pressure to participate.

 3. The manuscript would benefit from slightly clearer terminology regarding "participation rate," "response rate," "accessed the survey," "partial completion," and "full completion." These terms should be used consistently throughout the abstract, results, and discussion.

 4. Given the small numbers in some program groups, the authors should ensure that all demographic reporting sufficiently protects participant confidentiality, especially where categories contain very small cell counts.

 5. The limitations should more explicitly state that the high response rate may reflect the specific institutional context: a small private university, health professional students, scheduled class-time completion, and a strong student engagement culture. Therefore, the approach may not generalize directly to larger public universities or less structured teaching contexts.

 Overall, the manuscript is acceptable as a methodological report, but I recommend approval with reservations. The remaining issues do not fundamentally undermine the article, but addressing them would improve clarity, interpretation, and generalizability.

Is the work clearly and accurately presented and does it cite the current literature?

Partly

If applicable, is the statistical analysis and its interpretation appropriate?

Yes

Are all the source data underlying the results available to ensure full reproducibility?

Yes

Is the study design appropriate and is the work technically sound?

Yes

Are the conclusions drawn adequately supported by the results?

Partly

Are sufficient details of methods and analysis provided to allow replication by others?

Partly

Reviewer Expertise:

Mental health and well-being • Clinical psychology • Positive psychology• Occupational health psychology • Cultural and cross-cultural psychology • Health psychology• Family and relationship studies • Trauma and resilience • Sleep and psychological health• Digital and cyberpsychology • Gender and psychology • Forensic psychology• Criminal psychology • Political psychology • Artificial intelligence and psychology

I confirm that I have read this submission and believe that I have an appropriate level of expertise to confirm that it is of an acceptable scientific standard, however I have significant reservations, as outlined above.

# Reviewer report: Reviewer response for version 3

**DOI:** 10.5256/f1000research.199248.r510559

**Published:** 2026-08-19

**Authors:** Hengki Yandri

**Affiliations:** 1 Institut Agama Islam Negeri Kerinci, Jambi, Indonesia

**Abstract.**

 Revise the aim to be more explicit. For instance: "This study sought to develop and implement an online survey... with the primary goal of achieving higher participation and response rates than previously reported, by employing a co-design and in-class administration strategy. " In the conclusions, focus on the methodological achievement rather than the implications of findings that are not presented in this paper.

**Introduction.**

 Add a brief paragraph explaining why this specific student population was chosen. Was it due to accessibility within the faculty, or because they are viewed as future health promoters whose own well-being is particularly critical?

 Clearly articulate the research gap. It is not just the low response rate, but the lack of studies that systematically test a combination of evidence-based strategies (like Javidan et al.'s recommendations) simultaneously. This is the study's original contribution.

 Remove the sentence "The survey data will be used to inform..." from the end of the introduction, as it describes a future goal, not the aim of the current methodological paper.

**Methods**

**Participants and Recruitment**

 Clarify how the student representatives were selected. Were they elected by their peers, appointed by faculty, or self-selected? This is important for the transparency of the co-design process.

**Recruitment (Continued)**

 State clearly whether students who were absent on the day of data collection were given an alternative opportunity to complete the survey. If not, this is a limitation that should be acknowledged.

**Data Analysis**

 Provide a more rigorous, a priori definition of "partial completion" in the methods section, rather than introducing it in the results. For example: "Partial completion was defined as completing all items in the demographic section (Section 1)."

 Appendices: Ensure the link to Multimedia Appendix 2 is active and accessible, as it contains crucial information for replicating the implementation of the Javidan strategies.

**Results**

**Anonymity**

 As suggested by reviewer Flinn, carefully review Table 4. For variables with very low frequencies (e.g., <5 in a cell), combine categories or report as <5 to protect participant identities, especially given that the institution and specific programs are named.

**Participation Bias**

 Acknowledge that in-class administration, while effective, may introduce an implicit pressure to participate, which could artificially inflate the response rate. This is an important point to discuss (more appropriate for the Limitations section).

**Discussion**

**Reinforce the Aim**

 Begin the discussion by reaffirming the methodological focus of the paper. Discuss the success of the recruitment strategies as the central finding.

**Preliminary Findings (Optional)**

 If you wish to provide a glimpse, you could add one brief, descriptive paragraph about the most basic finding (e.g., "Descriptively, the sample reported high levels of perceived stress..."). However, if a separate manuscript is planned for the full analysis, state this explicitly: "A comprehensive analysis of the health and well-being data is underway and will be presented in a separate publication." This manages reader expectations.

**Strengths and Limitations**

 Strengthen this section. Acknowledge the limitations more candidly: the limited generalizability (single, small, private institution), the potential for social desirability bias (filling out the survey in a classroom setting), and the lack of a power analysis for subgroup comparisons.

Is the work clearly and accurately presented and does it cite the current literature?

Partly

If applicable, is the statistical analysis and its interpretation appropriate?

Yes

Are all the source data underlying the results available to ensure full reproducibility?

Yes

Is the study design appropriate and is the work technically sound?

Yes

Are the conclusions drawn adequately supported by the results?

Partly

Are sufficient details of methods and analysis provided to allow replication by others?

Partly

Reviewer Expertise:

Spiritual Counseling, Counseling, Educational Psychology, and Mental Health

I confirm that I have read this submission and believe that I have an appropriate level of expertise to confirm that it is of an acceptable scientific standard, however I have significant reservations, as outlined above.

# Reviewer report: Reviewer response for version 2

**DOI:** 10.5256/f1000research.193546.r465772

**Published:** 2026-03-28

**Authors:** Alba Madrid Cagigal

**Affiliations:** 1 University of Galway, Galway, Ireland

Thank you for the opportunity to review this manuscript describing the development and implementation of the BOOST-Well survey, an online instrument designed to assess health behaviours and well-being among medical and allied health students at an Australian university. The authors report a response rate of 73.6% (partial completion) and 63.7% (full completion), which is substantially higher than rates reported in prior literature.

 Below, I highlight some issues for consideration to strengthen the manuscript:

**1. Insifficient justification for the study and unclear novelty**. While the introduction highlights limitations of existing surveys (e.g., low response rates), it does not clearly articulate why a new survey was required, and what specifically differentiates the BOOST-Well survey from existing approaches.

Recommendation: The authors should explicity clarify what the novel contribution is, why existing surveys were insufficient for their context, and what gap this study fills beyong simply reporting higher response rates.

**2. Limited justification for use of survey methodology:** The manuscript assumes surveys are the appropriate method without justification.

Recommendation: Include a brief rational for why a survey is appropriate for captiring multidimensional health and wellbeing data, and why alternative approaches (e.g., interviews) were not suitable.

**3. Some methodological details are missing or unclear.** In terms of the timing of data collection, why were surveys administered at two different time points (May and September)? were there differences between cohorts? No justification or rationale is provided for sample size; while a formal power calculation may not be required for a descriptive methodological study, this should be explicitly stated.

**4. Potential participation bias**. Although the authors state that steps were taken to minimise coercion, administering the survey during scheduled class time may introduce implicit pressure to participate.

Recommendation: More explicitly acknowledge this as a limitation, and discuss how this may have influenced participation rates and the generalisability of findings.

**5. Interpretation and clarity of response rate comparisons**. The discussion contains unclear phrasing when comparing response rates with prior studies. The manuscript states that previous studies had “higher response rates” (22% and 31%), which are in fact lower than those reported here.

**6. Limited discussion of survey findings**. While the stated aim is methodological, the manuscript provides almost no discussion of the actual health and well-being data collected. This creates a disconnect, as readers are told the data will inform interventions, but no insight is provided into what was found.

Recommendation: Include at least a brief summary of key findings, or a claer statement that these findings will be reported separately in another manuscript. 

**7. Minor issues**: Minor wording issue: “Skewed variables are reported as (medians with IQR)”, parentheses unnecessary. Ensure consistency of terms such as "response rate", "completion rate", "participation rate"; these are currently used somewhat interchangeably. The manuscript claims the survey was kept “as short as possible,” yet includes 136 items. This tension should be acknowledged and justified more clearly.

Is the work clearly and accurately presented and does it cite the current literature?

Yes

If applicable, is the statistical analysis and its interpretation appropriate?

Yes

Are all the source data underlying the results available to ensure full reproducibility?

Partly

Is the study design appropriate and is the work technically sound?

Yes

Are the conclusions drawn adequately supported by the results?

Yes

Are sufficient details of methods and analysis provided to allow replication by others?

Partly

Reviewer Expertise:

University students mental health and digital mental health

I confirm that I have read this submission and believe that I have an appropriate level of expertise to confirm that it is of an acceptable scientific standard, however I have significant reservations, as outlined above.

# Reviewer report: Reviewer response for version 2

**DOI:** 10.5256/f1000research.193546.r461071

**Published:** 2026-03-05

**Authors:** Clodagh Flinn

**Affiliations:** 1 University College Dublin, Dublin, Ireland; 2 Psychology, Carleton University (Ringgold ID: 6339), Ottawa, Ontario, Canada

Thank you for your work revising this manuscript.

**Introduction**:

 Point 1: Why did the authors choose to examine medical and allied health students specifically, as opposed to university students more broadly? Building this rationale into the introduction would be useful.

 Point 2: In the last paragraph it says, "The survey data will be used to inform the development of evidence-based and targeted strategies for improvement of students’ health and well-being..."

 Given that the actual survey data is not discussed or analysed in this manuscript, I think this should be removed. The aim of *this *manuscript vs. the survey itself is summed up well in the first sentence in the discussion but should be clearly distinguished in the introduction too.

**Method**:

 Point 3: How exactly were potential participants invited to take part? During class time?

 Point 4: There were 273 students registered. Do you know how many were present on the data collection days? Were students who were absent on those days given the chance to complete the survey at a later date?

 (If participants completed the survey during class time only).

**Results**:

 Point 5: "partial" survey completion in vague. Please be specific here - for example, did participants fill in 90% of the survey or stop after the demographics?

 Please also update the abstract if needed. 

 Point 6: Thank you for updating the tables in response to my previous comment. 

 Can you please review table 4 in full to ensure changes are made throughout? E.g., some very low frequencies remain in the gender and country of birth rows (although not all are technically <5%, there are very few - less than 5 - participants).

 You could consider combining the OT and N&D groups - although I understand this is not ideal, it would avoid very small frequencies and help to protect the identities of participants.

**Discussion**:

 Point 7: Strengths and limitations. I think reframing the strength regarding the student-informed survey would be beneficial. As it stands, this section reads as though the actual content included in the survey is the strength, but the actual survey data isn't what this manuscript examined.

 I think that the student engagement in the study design could be reframed as a key methodological strength - PPI in research is so important and a major strength of this study - don't underplay it! Also discuss the integration of relevant faculty, etc. in the survey -this adds knowledge and feedback from a range of experts. 

**Minor points**:

 Introduction. Paragraph 2 begins with, "Unfortunately, health and well-being information about university students is typically obtained through surveys." 

 Consider rephrasing this sentence - there is nothing fundamentally wrong with collecting data with surveys, although there are challenges limitations (like with any methodology) which are discussed. 

 Methods. Add citation for statistical software, Jamovi.

 The link to appendix 2 does not appear to be active.

Is the work clearly and accurately presented and does it cite the current literature?

Partly

If applicable, is the statistical analysis and its interpretation appropriate?

Yes

Are all the source data underlying the results available to ensure full reproducibility?

Yes

Is the study design appropriate and is the work technically sound?

Yes

Are the conclusions drawn adequately supported by the results?

Yes

Are sufficient details of methods and analysis provided to allow replication by others?

No

Reviewer Expertise:

Psychological wellbeing and resilience. Chronic health conditions. Developmental transitions.

I confirm that I have read this submission and believe that I have an appropriate level of expertise to confirm that it is of an acceptable scientific standard, however I have significant reservations, as outlined above.

# Reviewer report: Reviewer response for version 1

**DOI:** 10.5256/f1000research.185745.r416932

**Published:** 2025-10-10

**Authors:** Clodagh Flinn

**Affiliations:** 1 University College Dublin, Dublin, Ireland; 2 Psychology, Carleton University (Ringgold ID: 6339), Ottawa, Ontario, Canada

The study aimed to develop an online survey on health, health behaviours and wellbeing for university students, implementing Javidan et al.'s recommendations in order to maximise response/participation rates.

 The collaborative development of the survey with students is a clear strength of the study, it is great to see this being actively integrated into the work.

 Overall, I believe this is a good study which addresses an important area - dealing with low response rates for research on student wellbeing.

 As it stands, the introduction/methods suggest that the main aim is to explore health and wellbeing, not to design and implement a survey. Based on the intro/methods, I expected to see findings of the actual survey in the results, e.g., are students reporting depressive symptoms, high levels of smoking, etc. I believe the aims may be somewhat confusing to readers - is it to develop a survey and boost/report response rates, or to investigate health and wellbeing in students?

 I think that the implementation of recommendations and strategies to increase participation are very important and central to the study, and so this should be highlighted as the primary main aim. This is a paper on the development of a survey and how you increased participation, but I did not feel that this was clear until the results/discussion.

 Clearly stating your aims and structuring your results accordingly would be useful. You could also consider splitting results into the initial pilot study (in 2022) and the main study (2024).

 Lastly, although this study is focused on how the researchers developed a survey and implemented a range of measures in order to increase participation, there is little description of this process in the methods section.

**Introduction:**

 -Given that this is a methodological/technical paper (survey development and implementation), the introduction focuses more on the general literature on student mental health. Because of this I expected to read about the actual health of the sample, and not only the response rates, etc.

**Method:**

 -The paper is about the development and implementation of a survey - there should be more detailed information on how exactly this was done.

 -Add more detail into the method about which topics specifically the survey covered, why were they chosen (e.g., based on the pilot study). How exactly was the survey developed?

 -More information should be included regarding specific measures, and especially the development of questions by the researchers. Why were certain measures used? -Were questions developed by the researchers based on previous research or the pilot study?

 -More detail on recruitment is needed - e.g., how did authors work with student/staff representatives? How were the representatives selected? What recruitment ideas were used from previous research? How were students invited to take part?

 -What are Javidan's recommendations and how were they implemented? (Given the centrality of maximising participation to the research, I expected to see more about how this was done).

 -Add explicit ethical acknowledgement into the methods section unless there is a reason not to.

**Results:**

 -Table 1 is very useful for showing exactly what was included in the survey. I think it should be moved up into the method section as it is helpful for contextualising the study.

 -There are some very small frequencies reported in the demographics (e.g., one participant responded "other" for gender within the occupational therapy group). I am concerned that some participants could be identified by this given the name of the research institution and area of study are available. Please check that this cannot occur, and consider changing smaller frequencies to <5% instead. 

**Discussion:**

 -There is more information about the survey development/recruitment in the discussion than the methods, but this is not where it should be encountered for the first time. Much of this should be moved to the methods section - e.g., procedures, incentives, etc. used in the present study.

 The discussion could consider why these initiatives worked, etc.

 -Could further develop the point about how the findings can be used for teaching purposes - you could also discuss how these implications go beyond the classroom, expanding them to broader researchers/policy makers too. This study is a great example of how response rates can be significantly increased, possibly yielding more representative samples, etc.

**Very minor points:**

 - Some very minor grammatical error (e.g., abstract: "meaning the representative of such data") - please do a thorough proof read.

 - Please include full explanations for all abbreviations within the tables.

Is the work clearly and accurately presented and does it cite the current literature?

Partly

If applicable, is the statistical analysis and its interpretation appropriate?

Yes

Are all the source data underlying the results available to ensure full reproducibility?

Yes

Is the study design appropriate and is the work technically sound?

Yes

Are the conclusions drawn adequately supported by the results?

Yes

Are sufficient details of methods and analysis provided to allow replication by others?

No

Reviewer Expertise:

Psychological wellbeing and resilience. Chronic health conditions. Developmental transitions.

I confirm that I have read this submission and believe that I have an appropriate level of expertise to confirm that it is of an acceptable scientific standard, however I have significant reservations, as outlined above.

# Reviewer report: Reviewer response for version 1

**DOI:** 10.5256/f1000research.185745.r407853

**Published:** 2025-09-16

**Authors:** Ali Azeez Al-Jumaili

**Affiliations:** 1 Department of Clinical Pharmacy, University of Baghdad, Baghdad, Baghdad Governorate, Iraq

**Reflections on the development and implementation of a university student health and well-being online survey: the BOOST-Well project**

**Reviewer Comments**

**Abstract**

 – The abstract lacks clarity and should be rewritten to clearly reflect the study’s aim, methodology, key findings, and conclusions.

 – Please remove citations from the abstract, as referencing other studies is not appropriate in this section.

**Methods**

 – The methodology section is currently vague. It should explicitly state the study design (e.g., cross-sectional survey), the setting (e.g., university faculty), the country of study (Australia), and the data collection period (month/year).

 – Clarify how participants were selected and provide justification for the sample size.

**Results**

 – Please specify the academic level of the student participants (e.g., undergraduate, postgraduate).

 – The results section lacks substantive findings. Include key outcomes supported by descriptive statistics or thematic summaries.

 – Participant demographics and characteristics should be clearly presented to contextualize the findings.

**Introduction**

 – The stated aim is overly detailed and should be streamlined. Consider summarizing the aim in one concise sentence.

 – Clarify whether the study has one primary aim or multiple objectives, and ensure these are aligned with the abstract and methods sections.

**Methods**

 Could you provide a completed CROSS checklist as supplementary material or clarify which specific items were most influential in shaping your survey design and reporting?

 Were any psychometric properties (e.g., reliability, construct validity) assessed for the BOOST-Well survey items?

 Why were only three student leaders included in the pilot phase? Would broader pilot testing have helped capture more diverse feedback?

 How was the sample size determined to ensure adequate power for subgroup comparisons?

 Were any demographic groups underrepresented in the final sample, and if so, how might this affect the generalizability of your findings?

 How were missing or incomplete responses handled during analysis, particularly for questions with revised instructions (e.g., physical activity)?

  Which specific variables were marked as “NR” due to unmet assumptions, and how did this affect interpretation of group comparisons?

 Why was Jamovi selected for analysis, and were any limitations encountered compared to other platforms like SPSS or R?

**Results**

 Table 2-It is better to separate results those with NR from those with P-value (using different tables)

**Table 4 **seems part of review article summarizing previous studies.

 Please justify why your research includes such a summary, given it is a research article.

**Conclusion**

 “ *An online survey was developed to better understand the health behaviors and health and well-being for medical and allied health students, with a completion rate that is substantially higher than that typically reported.”*

 This sentence is not supported by results (at least they are not shown in the abstract results).

Is the work clearly and accurately presented and does it cite the current literature?

No

If applicable, is the statistical analysis and its interpretation appropriate?

Partly

Are all the source data underlying the results available to ensure full reproducibility?

Partly

Is the study design appropriate and is the work technically sound?

Partly

Are the conclusions drawn adequately supported by the results?

No

Are sufficient details of methods and analysis provided to allow replication by others?

No

Reviewer Expertise:

Social and Administrative Pharmacy, Pharmacy Education, Pharmacy Practice, Clinical Pharmacy, Public Health, and Pharmacoeconomics.

I confirm that I have read this submission and believe that I have an appropriate level of expertise to state that I do not consider it to be of an acceptable scientific standard, for reasons outlined above.

# Response

**Published:** 2025-12-15

**Authors:** JUSTIN Keogh

**Affiliations:** Bond University Faculty of Health Sciences and Medicine, Gold Coast, Queensland, Australia

**Reflections on the development and implementation of a university student health and well-being online survey: the BOOST-Well project**

**Reviewer Comments**

**Abstract**

 – The abstract lacks clarity and should be rewritten to clearly reflect the study’s aim, methodology, key findings, and conclusions.

 – Please remove citations from the abstract, as referencing other studies is not appropriate in this section.

**Response: we have rewritten the abstract to more closely align with the intended aim of the study i.e. developing a health and well-being survey methodology that increases response rates in university students. We have also removed the reference from this section as requested**

**Methods**

 – The methodology section is currently vague. It should explicitly state the study design (e.g., cross-sectional survey), the setting (e.g., university faculty), the country of study (Australia), and the data collection period (month/year).

 – Clarify how participants were selected and provide justification for the sample size.

**Response: some additional detail regarding the study design, country and collection period has been provided. However, we would like to reiterate that some of this information regarding the setting, how participants were selected and sample size had already been provided in the initial submission.**

**Results**

 – Please specify the academic level of the student participants (e.g., undergraduate, postgraduate).

 – The results section lacks substantive findings. Include key outcomes supported by descriptive statistics or thematic summaries.

 – Participant demographics and characteristics should be clearly presented to contextualize the findings.

**Response: The method section had stated that participants were undergraduate medical and postgraduate allied health students. Additional demographic characteristics was attempted to be added in the results section.  however, this request and other requests that the abstracts exceeded 400 words, much beyond the 300 word maximum capacity for an abstract to this journal. Hence,  these demographic details cannot be included. In regards to the lack of reporting of key outcomes, that may reflect a lack of clarity regarding the focus of the paper, which is now been rewritten in the revised manuscript to be “The primary aim of this study was therefore to develop and implement an online survey to examine the health and well-being behaviours of medical and allied health students, with higher participation and response rate than have been reported in previous surveys.”. As such, reporting on the actual health and well-being survey results was not a focus of this paper; with such results to be presented in additional follow-on manuscripts.**

**Introduction**

 – The stated aim is overly detailed and should be streamlined. Consider summarizing the aim in one concise sentence.

 – Clarify whether the study has one primary aim or multiple objectives, and ensure these are aligned with the abstract and methods sections.

**Response: The primary aim has been rewritten to “The primary aim of this study was therefore to develop and implement an online survey to examine the health and well-being behaviours of medical and allied health students, with higher participation and response rate than have been reported in previous surveys.”. The secondary aim of developing a resource for students to develop their research skills has been retained; with some discussion of this component still retained in the manuscript.**

**Methods**

 Could you provide a completed CROSS checklist as supplementary material or clarify which specific items were most influential in shaping your survey design and reporting?

**Response: we thank the reviewer for this comment and have provided some additional detail in the methodology and result sections of the manuscript in regard to how they more explicitly addressed requirements of the CROSS checklist and the limitations identified by Bennett et al.** **[1]** **survey based medical research.**

**In a systematic review of 165 medical journals regarding survey-based research, Bennett et al.** **[1]** **identified a number of major methodological issues regarding survey-based research. Of the 117 published survey studies they reviewed, only 35% of studies provided the survey or core questions, 19% provided any data on the validity or reliability of the instruments, 25% defined the response rate, 11% discussed the representativeness of the sample and 11% identified how missing data were handled. To address these issues identified by Bennett et al.** **[1]** **, the most important areas of the CROSS checklist that were influential in shaping our survey design are described below.**

 Data collection methods

 5a

 Describe the questionnaire (e.g., number of sections, number of questions, number and names of instruments used).

 5b

 Describe all questionnaire instruments that were used in the survey to measure particular concepts. Report target population, reported validity and reliability information, scoring/classification procedure, and reference links (if any).

 5d

 Questionnaire if possible, should be fully provided (in the article, or as appendices or as an online supplement).

 Sample characteristics

 6a

 Describe the study population (i.e., background, locations, eligibility criteria for participant inclusion in survey, exclusion criteria).

 6d

 Describe how representative the sample is of the study population (or target population if possible), particularly for population-based surveys.

 Survey

 administration

 7a

 Provide information on modes of questionnaire administration, including the type and number of contacts, the location where the survey was conducted (e.g., outpatient room or by use of online tools, such as SurveyMonkey).

 7b

 Provide information of survey’s time frame, such as periods of recruitment, exposure, and follow-up days.

 Were any psychometric properties (e.g., reliability, construct validity) assessed for the BOOST-Well survey items?

**Response: The current Table 1 which is found in the results provides references for all the validated and commonly used questionnaires utilised to examine the topics included in the survey which include: quality of life; time use, stress and social support; health behaviours e.g. physical activity, fruit and vegetable consumption etc; and health services and medications. Table 1 also provides references for the demographic characteristics used. In our approach we adapted the questions from a range of validated health surveys. This table and some additional detail regarding these assessments and the rationale for their choice has been provided in a text section in the revised method section.**

 Why were only three student leaders included in the pilot phase? Would broader pilot testing have helped capture more diverse feedback?

**Response: Student leaders were the specific leaders of the three medical and allied health groups that represented our participants. As such, these three leaders attended meetings as representatives of their degree cohorts and of the Community Health and Well-Being Officer. Prior to this we also posted a survey to selected individuals across these programs in which 45 students responded regarding what they thought the five main health and well-being issues/topics should be. Mention of this was provided in the third paragraph of the discussion within the initial version of the manuscript, although we realise that should have been included in the methods, with this revision now made in the revised manuscript.**

 How was the sample size determined to ensure adequate power for subgroup comparisons?

**Response: as the focus of this study was to develop a survey methodology that increases response rates, it was felt that a power analysis for subgroup comparisons was not required. Further, as stated in the Participants and Recruitment sub-section within the Materials and Methods section, “All new and enrolled students (defined as commencing a degree that semester) from the undergraduate medical and postgraduate allied health programs were invited to participate.”  Hence, we were unable to recruit any more participants than those who were starting those degrees in the recruitment semesters (this being 273 registered students). We have however noted in limitations to the study that no power analyses were performed for subgroup comparisons and that this may most affect comparisons with the nutrition and dietetics degree due to their smallest sample size.**

 Were any demographic groups underrepresented in the final sample, and if so, how might this affect the generalizability of your findings?

**Response: An inspection of the participants who fully completed the survey and those who either partially or failed to complete the survey did not reveal any major differences in their demographic characteristics. This suggests that the sample of participants who provided full responses was representative of the entire cohort.**

 How were missing or incomplete responses handled during analysis, particularly for questions with revised instructions (e.g., physical activity)?

**Response: Questions that subsequently had minor revisions like program of study and language spoken at home either did not have any missing data after data cleaning or had very low missing data (0.5%) that were not used in analysis. Physical activity responses are covered in a separate piece of work being considered for publication and follow a series of data cleaning and processing that handle missing or incomplete data in a systematic way following the methods of Brown et al.** **[2]** **that were cited in the original article.**

  Which specific variables were marked as “NR” due to unmet assumptions, and how did this affect interpretation of group comparisons?

**Response: The variables marked as NR for unmet assumptions were all the ones reported “NR” in the p-value column in Table 2 (now Table 4), apart from ‘Indigenous origin’ where testing was not applicable as the responses were always ‘No’, if not missing. We did not rely on hypothesis testing in our interpretation of group comparisons that were mostly descriptive, and have therefore removed the *p*-value column, and mentioned any significant differences tested in the text instead.**

 Why was Jamovi selected for analysis, and were any limitations encountered compared to other platforms like SPSS or R?

**Response: there were no limitations encountered using Jamovi compared to SPSS or R in this study. Jamovi conveniently combines both a familiar interface for SPSS users, as well as the features of R since it incorporates commonly used R packages as well as R packages for more complex analyses all under one software that is very user-friendly. Two of the authors (Keogh and Rathbone) have successfully utilised Jamovi as being statistical analysis package in several other of their recent peer-reviewed journal publications. Examples include:**

**https://pmc.ncbi.nlm.nih.gov/articles/PMC9135035/**

**https://pmc.ncbi.nlm.nih.gov/articles/PMC10959102/**

**https://pubmed.ncbi.nlm.nih.gov/35651742/**

**https://www.pathologyjournal.rcpa.edu.au/article/S0031-3025(23)00076-4/fulltext**

**https://link.springer.com/article/10.1007/s00586-025-08926-6**

**Results**

 Table 2-It is better to separate results those with NR from those with P-value (using different tables)

**Response: Table 2 (now Table 4) gives a snapshot of demographic characteristics, so we can compare all the variables, mostly in a descriptive way and this type of format is common in survey results reporting. However, if we split the tables, we would be putting more emphasis on *p*-values which have only been added where possible to complement the essential statistics.**

**As stated above, we have now removed the *p*-value column so that the focus is on descriptive statistics, whilst we have summarised the results of a couple of significance tests in the main text**

**Table 4 **seems part of review article summarizing previous studies.

 Please justify why your research includes such a summary, given it is a research article.

**Response: based on the good points from the reviewer to make it more explicit that this manuscript focuses on the development of a survey that increases response rates, this summary table and some of the associated text has now been moved to the introduction to further support the importance of this study.**

**Conclusion**

 “ *An online survey was developed to better understand the health behaviors and health and well-being for medical and allied health students, with a completion rate that is substantially higher than that typically reported.”*

 This sentence is not supported by results (at least they are not shown in the abstract results).

**Response: we acknowledge the way this has been interpreted and have changed the sentence to “By following a *series of recommendations from the literature, we developed an online health and well-being survey that had a completion rate that is substantially higher than that typically reported for other studies involving university students*”.**

 Reviewer Report

 1 Views

 copyright

*10 Oct 2025 | for Version 1*

**Clodagh Flinn**, University College Dublin, Dublin, Ireland;  Psychology, Carleton University (Ringgold ID: 6339), Ottawa, Ontario, Canada 

format_quote Cite this report speaker_notes Responses(0)

  Approved With Reservations

 info_outline

 The study aimed to develop an online survey on health, health behaviours and wellbeing for university students, implementing Javidan et al.'s recommendations in order to maximise response/participation rates.

 The collaborative development of the survey with students is a clear strength of the study, it is great to see this being actively integrated into the work.

 Overall, I believe this is a good study which addresses an important area - dealing with low response rates for research on student wellbeing.

 As it stands, the introduction/methods suggest that the main aim is to explore health and wellbeing, not to design and implement a survey. Based on the intro/methods, I expected to see findings of the actual survey in the results, e.g., are students reporting depressive symptoms, high levels of smoking, etc. I believe the aims may be somewhat confusing to readers - is it to develop a survey and boost/report response rates, or to investigate health and wellbeing in students?

 I think that the implementation of recommendations and strategies to increase participation are very important and central to the study, and so this should be highlighted as the primary main aim. This is a paper on the development of a survey and how you increased participation, but I did not feel that this was clear until the results/discussion.

**Response: we thank the reviewer for these comments and have now altered the Introduction, Method and Results sections to be more closely aligned with the developmental focus of this study in regards to improving response rates for student health and well-being surveys.**

 Clearly stating your aims and structuring your results accordingly would be useful. You could also consider splitting results into the initial pilot study (in 2022) and the main study (2024).

**Response: we have restated the aims and the structure of the results to make it clearer that this manuscript describes the development of the survey approach and focuses its results on the response rates and associated outcomes relevant to improving response rates in similar samples of university students.**

 Lastly, although this study is focused on how the researchers developed a survey and implemented a range of measures in order to increase participation, there is little description of this process in the methods section.

**Response: additional information is now provided regarding the development of the survey in the methods section.**

**Introduction:**

 -Given that this is a methodological/technical paper (survey development and implementation), the introduction focuses more on the general literature on student mental health. Because of this I expected to read about the actual health of the sample, and not only the response rates, etc.

**Response: the emphasis of introduction has now been adapted as requested to focus more on survey response rates and associated methodological concerns rather than student health and well-being.**

**Method:**

 -The paper is about the development and implementation of a survey - there should be more detailed information on how exactly this was done.

**Response: additional detail has now been provided in the revised methods and result sections regarding the development and implementation of the survey.**

 -Add more detail into the method about which topics specifically the survey covered, why were they chosen (e.g., based on the pilot study). How exactly was the survey developed?

**Response: additional detail regarding the process by which specific topics were identified by the researchers and students as being most important from the pilot study have also now been provided.**

 -More information should be included regarding specific measures, and especially the development of questions by the researchers. Why were certain measures used? -Were questions developed by the researchers based on previous research or the pilot study?

**Response: the current Table 1 which is found in the Results section provides references for all the validated and commonly used questionnaires utilised to examine all of the important topics included in the survey which include: quality of life; time use, stress and social support; health behaviours e.g. physical activity, fruit and vegetable consumption etc; and health services and medications. Table 1 also provides references for the references for the demographic characteristics that were typically slightly adapted from a range of validated health surveys. This table and some additional detail regarding these assessments and the rationale for their choice has been provided in a text section in the revised method section.**

 -More detail on recruitment is needed - e.g., how did authors work with student/staff representatives? How were the representatives selected? What recruitment ideas were used from previous research? How were students invited to take part?

**Response: additional detail regarding this point has now been added to the revised methods.**

 -What are Javidan's recommendations and how were they implemented? (Given the centrality of maximising participation to the research, I expected to see more about how this was done).

**Response: an additional table and text describing the six recommendations has now been added to the method section.**

 -Add explicit ethical acknowledgement into the methods section unless there is a reason not to.

**Response: the ethical acknowledgement section has been reinstated into the method section.**

**Results:**

 -Table 1 is very useful for showing exactly what was included in the survey. I think it should be moved up into the method section as it is helpful for contextualising the study.

**Response: We agree with this request and would normally do this.  However, since we have also included the response rate to each of the different questionnaire items in this table, we feel it is better having all of this included in the results section. Repeating this entire table with the response rate information in the results section would therefore appear redundant.**

 -There are some very small frequencies reported in the demographics (e.g., one participant responded "other" for gender within the occupational therapy group). I am concerned that some participants could be identified by this given the name of the research institution and area of study are available. Please check that this cannot occur, and consider changing smaller frequencies to <5% instead. 

**Response: Data about students from individual programs were only available to the lead data custodian who also analysed them but was not involved with any of the cohorts. Prior to issuing data to other researchers, the data from the physiotherapy, occupational therapy and nutrition and dietetics groups were combined to create the allied health group, so that any such identification cannot occur. We appreciate the suggestion to change the smaller frequencies; as an additional precaution, we have combined the ‘Prefer not to say’ and ‘Other’ categories within Gender into one and replaced the low counts within the individual programs with ‘<5%’.**

**Discussion:**

 -There is more information about the survey development/recruitment in the discussion than the methods, but this is not where it should be encountered for the first time. Much of this should be moved to the methods section - e.g., procedures, incentives, etc. used in the present study.

**Response: this has been done as requested.**

 The discussion could consider why these initiatives worked, etc.

 -Could further develop the point about how the findings can be used for teaching purposes - you could also discuss how these implications go beyond the classroom, expanding them to broader researchers/policy makers too. This study is a great example of how response rates can be significantly increased, possibly yielding more representative samples, etc.

**Response: additional detail has been provided regarding the wider implications of these findings as requested.**

**Very minor points:**

 - Some very minor grammatical error (e.g., abstract: "meaning the representative of such data") - please do a thorough proof read.

 - Please include full explanations for all abbreviations within the tables.

**Response: an additional proofread and all explanations underneath tables have been checked for abbreviations.**

**References included in this response document**

 1.         Bennett C, Khangura S, Brehaut JC, Graham ID, Moher D, Potter BK, et al. Reporting guidelines for survey research: An analysis of published guidance and reporting practices. PLOS Medicine. 2010;8(8):e1001069. doi: 10.1371/journal.pmed.1001069.

 2.         Brown WJ, Burton NW, Marshall AL, Miller YD. Reliability and validity of a modified self-administered version of the Active Australia physical activity survey in a sample of mid-age women. Aust N Z J Public Health. 2008;32(6):535-41. PMID: 19076744. doi: 10.1111/j.1753-6405.2008.00305.x.
